# Cellodin as an Embedding Mass

**Published:** 1884-08

**Authors:** Boerne Betterman

**Affiliations:** 113 Adams Street; Chicago


ARTICLE II.

### CELLOIDIN AS AN EMBEDDING MASS. *By* DR. BOERNE BETTMAN, *Chicago.*

[*Read before the State Microscopical Society, June* 13, 1884.]

Not very long ago I exhibited to the members of this society an improved microtome and samples of embedding masses. Since then I have had occasion to employ a preparation but lately made known to the scientific world, which surpasses in every respect those heretofore in vogue. This preparation is known as Celloidin mass.

Celloidin is the purest gun cotton or pyroxylin. It is manufactured by Mr. G. Schering, of Berlin. He has succeeded in making, by a patented method, an article free from all precipitating organic substances, which, in distinction from all other gun cotton, he calls by this name. Its use in the form of gelatinous plates is, according to the manufacturer's statements, free from danger, it being inexplosive. The mass in question was first employed by Prof. Otto Becker, of Heidelberg, who pays a high tribute to its excellent qualities in his classical work, "The Pathology of the Lens."

It is prepared in the following manner: Take of alcohol and sulphuric ether equal parts, mix and add sufficient celloidin to form a jelly-like substance. The preparation must be kept in an air-tight vessel. The specimen to be embedded should have undergone the usual hardening process, in Müller's fluid and alcohol; it is then stained in bulk by carmine, hæmatoxylin, or any other tincture. After having taken up sufficient coloring matter, which is usually accomplished in 24 hours, the specimen should be thorougly cleansed by immersing it in running water. Then it is placed in absolute alcohol 24 hours longer. It is now ready for the celloidin bath, where it is permitted to remain at least 24 hours, in order that the mass may permeate the tissues thoroughly. If the cover of the jar containing the embedding mass is a close fitting one, preventing the evaporation of the ether and alcohol, the specimen may remain for an indefinite period.

The final step is the pasting of the preparation on a cork. A few moments' exposure to the air will suffice to fasten the specimen sufficiently firmly to allow of its immersion in methylated spirit, sp. gr. 82. It should be exposed to the hardening action of alcohol from 15 to 24 hours before being cut.

In the majority of cases it is desirable to mount the section encased in the celloidin. This necessitates a slight deviation from the course ordinarily pursued. Oleum origanum, or bergamot oil must be substituted for clove oil. Bergamot oil is preferable to the former. If origanum oil be employed, the preparation should be transported rapidly ; an exposure longer than one minute to this oil will produce visible changes in the celloidin, it becomes cloudy, and impairs the value of the cut. In other respects the method of treating the sections is the usual one.

A few moments' exposure to clove oil dissolves the mass.

The advantages of this preparation over others are manifold. The material employed is clean, experience forms no factor in its preparation, which is facile and rapid. It permeates the tissues thoroughly, and fixes the various parts in position, thus preserving their relationship. It is perfectly transparent, amorphous, and, since the preparation to be embedded can be stained beforehand, it remains invisible to the eye, and does not detract from the usefulness and appearance of the section.

113 Adams Street.

